# Model of genetic and environmental factors associated with type 2 diabetes mellitus in a Chinese Han population

**DOI:** 10.1186/s12889-020-09130-5

**Published:** 2020-06-29

**Authors:** Zheng Li, Cheng-yin Ye, Tian-Yu Zhao, Lei Yang

**Affiliations:** 1 Medical School, Hangzhou Normal University, 2318 Yuhangtang Rd, Hangzhou, 310000 Zhejiang China; 2 Medical School, Shihezi University, Shihezi, 832000 China

**Keywords:** Type 2 diabetes mellitus, Elastic net, Single-nucleotide polymorphism, Environmental factors

## Abstract

**Background:**

Type 2 diabetes mellitus (T2DM) is a metabolic disorder which accounts for high morbidity and mortality due to complications like renal failure, amputations, cardiovascular disease, and cerebrovascular events.

**Methods:**

We collected medical reports, lifestyle details, and blood samples of individuals and used the polymerase chain reaction-ligase detection reaction method to genotype the SNPs, and a visit was conducted in August 2016 to obtain the incidence of Type 2 diabetes in the 2113 eligible people. To explore which genes and environmental factors are associated with type 2 diabetes mellitus in a Chinese Han population, we used elastic net to build a model, which is to explain which variables are strongly associated with T2DM, rather than predict the occurrence of T2DM.

**Result:**

The genotype of the additive of rs964184, together with the history of hypertension, regular intake of meat and waist circumference, increased the risk of T2DM (adjusted OR = 2.38, *p* = 0.042; adjusted OR = 3.31, *p* < 0.001; adjusted OR = 1.05, *p* < 0.001). The TT genotype of the additive and recessive models of rs12654264, the CC genotype of the additive and dominant models of rs2065412, the TT genotype of the additive and dominant models of rs4149336, together with the degree of education, regular exercise, reduced the risk of T2DM (adjusted OR = 0.46, *p* = 0.017; adjusted OR = 0.53, *p* = 0.021; adjusted OR = 0.59, *p* = 0.021; adjusted OR = 0.57, *p* = 0.01; adjusted OR = 0.59, *p* = 0.021; adjusted OR = 0.57, *p* = 0.01; adjusted OR = 0.50, *p* = 0.007; adjusted OR = 0.80, *p* = 0.032) .

**Conclusion:**

Eventually we identified a set of SNPs and environmental factors: rs5805 in the *SLC12A3*, rs12654264 in the *HMGCR*, rs2065412 and rs414936 in the *ABCA1*, rs96418 in the *ZPR1* gene, waistline, degree of education, exercise frequency, hypertension, and the intake of meat. Although there was no interaction between these variables, people with two risk factors had a higher risk of T2DM than those only having one factor. These results provide the theoretical basis for gene and other risk factors screening to prevent T2DM.

## Background

As a global public health issue causing significant morbidity and mortality, type 2 diabetes mellitus (T2DM) affects more than 380 million people worldwide [1, 2]. The International Diabetes Federation has estimated that the number of individuals with diabetes will increase from 240 million in 2007 to 642 million in 2040 [3, 4]. In China, because of scientific and technological advances as well as socioeconomic development, the number of patients with diabetes is predicted to increase from 20.8 million in 2000 to 42.3 million in 2030 [5, 6]. China has the largest number of people with diabetes, with 92.4 million adults currently affected [7, 8]. T2DM accounts for approximately 90% of all diabetes cases, with an overall prevalence of 9.1% of the population. T2DM occurs mainly when the body becomes unable to effectively use insulin and pancreatic β cells to compensate for an enhanced insulin demand, leading to uncontrolled glucose homeostasis [2, 9]. Over time, poor glycemic control affects the blood vessels and nerves, accelerating the development and progression of neuropathies, micro- and macrovascular complications, and premature death [9, 10].

Most cases of T2DM are closely related to genetic and environmental risk factors [11, 12] and their interactions [13]. Previous genome-wide association studies [14–16] have identified numerous genetic polymorphisms and rare genetic variants associated with slight or significant effects on T2DM, suggesting that the disease results from complex interactions between genetic mechanisms and environmental factors. For instance, Zhang et al. [17] found a close relationship between the SLC12A3 gene and T2DM, and showed that a T allele in this gene had a modestly unfavorable impact on lipid levels. Ference et al. [18] showed that the genetic variants of the HMGCR gene are associated with T2DM. Ergen et al. [19] suggested ABCA1 polymorphism as a genetic marker of T2DM. Fumitaka et al. [20] identified the genetic susceptibility of patients with a novel common variant of rs964184 in ZPR1 to T2DM.

In addition to genetic predisposition, epidemiological risk factors play crucial roles in T2DM, such as gender differences, body mass index (BMI, weight in kilograms divided by height in square meters), lifestyle (e.g., smoking, alcohol consumption, etc.), and interactions between various factors [11–13, 21, 22].

We comprehensively analyzed the potential interactions between genes, physiological indices, biochemical indicators, and behavioral factors and T2DM. We constructed a model by elastic net that included genes and other environmental factors to identify variables strongly associated with T2DM rather than to predict the occurrence of T2DM.

## Methods

### Subjects

**Fig. 1:**
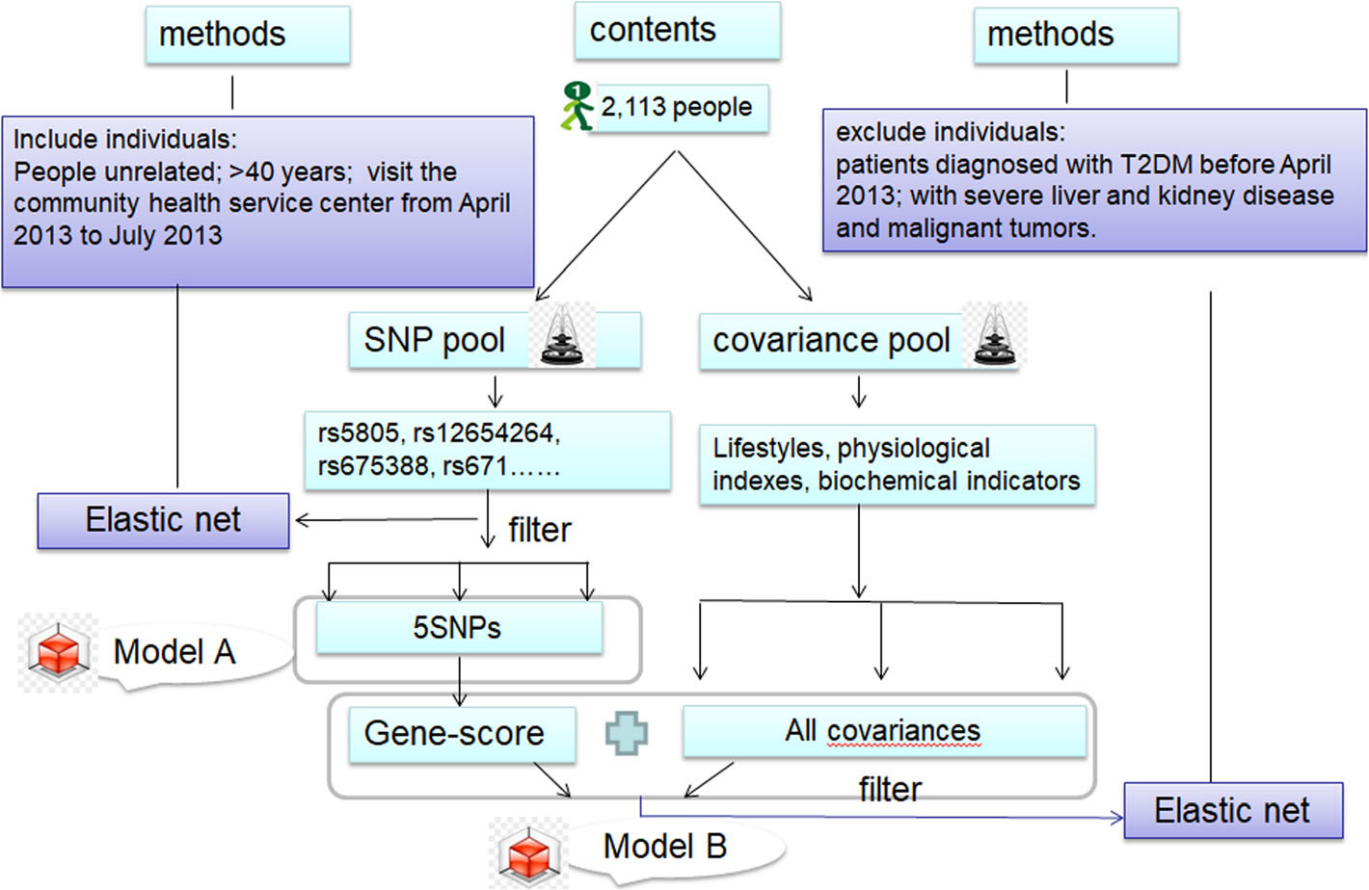
Study design

A total of 2323 subjects, who underwent physical examination at a community health service center from April 2013 to July 2013, were selected by cluster random sampling from 4 towns and townships in a district of Ningbo, Zhejiang Province. All subjects had to meet the following criteria: (1) Permanent residents aged more than 40 years old; (2) Han ethnic; (3) no consanguinity relation; (4) free from patients diagnosed with T2DM in April 2013, as well as patients with severe liver and kidney disease, malignant tumors and infectious diseases. We collected individual medical reports, lifestyle details, and blood samples and performed genotyping for single-nucleotide polymorphisms (SNPs) using the polymerase chain reaction-ligase detection reaction method. Interviews were performed in August 2016 to determine the subjects’ incidence of T2DM. A total of 2113 people qualified for the study. T2DM was diagnosed based on World Health Organization guidelines [23]. The case group included 100 patients diagnosed with CAD between April 2013 and August 2016. The rest who did not develop type 2 diabetes in 2016 were in the control group. The study was approved by the Medical Ethics Committee of Hangzhou Normal University (No. 2013020), all participants signed informed consent forms. The study design is as follows (Fig. 1):

### Demographic information and epidemiological investigation

Demographic variables mainly consisted of fundamental demographic criteria such as age, sex, education level and information on lifestyle such as smoking and drinking behavior. The main lifestyle variables were defined as follows. (1) Diet: “drink milk” and “drink soymilk” were defined as maintaining a certain amount of milk or soymilk intake every day, whereas “no milk intake habit” was defined as “not drinking”. An average intake of fried food of less than 1 time per week was defined as “no fried food”; those who ate less than one sweet treat per week were defined as “not eating sweets”. (2) Smoking: smoking behavior was defined as smoking at least one cigarette per day for at least 1 year. (3) Drinking: drinking behavior was defined as drinking white wine ≥50 g, red wine ≥150 g, or beer ≥500 g on average every day for 1 year or more. (4) Physical activity classification: “there is little physical activity, such as desk workers such as secretary” was defined as “sedentary”; “Light physical activity” was defined as “office work, repair of electrical clocks and watches, sales clerks, hotel services, chemical laboratory operations, lectures, etc.”; “Students’ daily activities, motor vehicle driving, electrical installation, lathe operation, metal cutting, etc.” was defined as “moderate physical activities”; “Non-mechanized agricultural labor, steelmaking, dancing, sports movement, loading and unloading, mining, loading and unloading cargo, construction workers, etc.” was defined as “heavy physical activity”.

Aaccording to standard protocols, anthropometric data, including weight, waist circumference, BMI, total cholesterol (TC), triglycerides (TG), high-density lipoprotein-cholesterol (HDL-C), and low-density lipoprotein-cholesterol (LDL-C) levels, systolic blood pressure (SBP), diastolic blood pressure (DBP) were evaluated by professional medical examinations.

Blood samples were collected from the antecubital vein after the subjects had fasted for ≥8 h. Part of the collected samples was used to examine biochemical indicators such as serum lipid levels, whereas the other part was transferred into a test tube containing anti-coagulant solution to extract DNA.

### Isolation of genomic DNA

Genomic DNA was extracted from the blood cells using a standard phenol/chloroform extraction method, centrifuged, and stored at − 80 °C. All genomic DNA samples were analyzed by electrophoresis. DNA was extracted using Tiangen Blood Genomic DNA extraction kits (Tiangen Biotech, Beijing, China) and sent to Shanghai Jierui Biological Engineering Co., Ltd., for genotyping analysis using the polymerase chain reaction (PCR)-ligase detection reaction (LDR) method (Generay Biotech Company, Shanghai, China). For this part, we have covered this in detail in previous articles [24]. For quality control, we randomly chose 10% of samples for re-genotyping, and the concordance was 100%.

### SNP selection and genotyping

Peripheral venous blood samples were collected from the study subjects to evaluate four physiological indicators of blood lipids (TC, TG, HDL-C, LDL-C and gene locus information. SNPs were mainly searched using the PubMed, Kyoto Encyclopedia of Genes and Genomes, and GeneCard databases. The specific screening process was as follows: (1) Literature related to gene polymorphisms, lipid levels, and atherosclerosis were searched in NCB-PubMed, and SNPs were screened; (2) GeneView information was obtained for relevant SNPs from the GeneCards database and NCBI database, and then, missense mutations, 3′ untranslated region (3′ UTR), 5′ UTR, or transcription factor-binding sites were selected; (3) The minor allele frequency (MAF) of SNPs in the Chinese population was detected from the HapMap database for the international human genome, and SNP sites with MAF values greater than 0.05 were screened; (4) Haploview software was used to conduct linkage imbalance analysis on all selected sites, and tagSNP was selected with *r*^*2*^ ≥ 0.8 as the standard.

This process identified 103 SNPs, including those in *SLC12A3*, *HMGCR*, *ABCA1*, and *KCNJ1*, among others. Information regarding all SNP loci is shown in Table S1.

### Statistical analysis

Statistical analysis was conducted with SPSS 24.0 software (SPSS, Inc., Chicago, IL, USA) and RStudio (Version 1.1.456. RStudio: Integrated development environment for R. Boston, MA, USA; http://www.rstudio.org/) using the glmnet package [25]. Elastic net regularization was used for feature selection which automatically performs variable selection to shrink the model to reduce over-fitting and co-variate correlation [26]. This technique has been shown to be superior to other methods of analysis when the set of features is much larger than the number of cases [27]. Chi-squared test, *t* test, Fisher exact test (for categorical variables), and Wilcoxon rank sum test (for continuous variables) were used to evaluate demographic characteristics and SNP genotypes. The odd ratios (ORs) and 95% confidence intervals (CIs) by logistic regression analysis were used to estimate the associations between variables (such as genetic models and lifestyles)and the risk of T2DM. The logistic-regression model based on 102 SNP feature selection and model based on SNP/ lifestyle features were separately developed on an elastic net. A gene-score was calculated for each person via the elastic net of 5 selected SNPs weighted by their respective coefficients. The gene-scores were combined with 31 environmental variables and 6 variables were screened out, including gene-scores with nonzero coefficients as determined by elastic net. Finally, receiver operating characteristic (ROC) curves were plotted to assess the efficiency of the model. Acoording to Knol [28], we used Excel software to identified interaction (RERI), OR, and 95% CI. Haploview, plink, and g-plink were used to calculate the *p* values of Hardy-Weinberg equilibrium. In all analyses, *p* values < 0.05 were considered to indicate a statistically significant difference. The purpose of this study was not to establish a model with good performance in predicting T2DM, but rather to explain T2DM through a relatively meaningful model, such as which SNP or environmental factors are likely to cause the disease.

## Results

### General characteristics

The subjects included 2163 randomly selected men and women: 54% of the subjects were female and 46% were male. A summary of their demographic characteristics such as age, sex, BMI, weight, HDL-C, LDC-C, TC, and TG is shown in Table 1. There were significant differences in age, weight, BMI, waistline, SBP, DBP, TG, LDL-C, degree of education, and exercise frequency between the case and control groups (*p* < 0.05) (Table 1). All studied SNPs in the control subjects were in Hardy-Weinberg equilibrium (*p* > 0.05). The MAF of each SNP was more than 5% to ensure that this study had sufficient statistical power (Table S1).

**Table 1:** Basic characteristics

Characteristic	T2DM (+)	T2DM (−)	t/z/χ2	P
Total	100	2013		
Age, Median (IQR), year	64(14.5)	62(17)	7.04	0.0080
Sex, n (%)			1.23	0.2680
Male	49(49)	917(45.5)		
Female	51(51)	1096(54.5)		
Weight, Median (IQR), year	63.2(5.05)	59.4(12.82)	5.49	0.0192
BMI, mean ± SD, kg/m2	24.06 ± 4.55	23.15 ± 3.84	8.32	0.0039
Waistline, Median (IQR), cm	82.87(11)		15.10	0.0001
SBP, Median (IQR), mmHg	145(16.5)	136(30)	23.80	< 0.001
DBP, Median (IQR), mmHg	90(15)	80(16)	17.27	< 0.001
TC, Median (IQR), mmol/L	4.99(1.39)	4.85(1.27)	1.99	0.1584
TG, Median (IQR), mmol/L	1.50 (0.65)	1.28(0.87)	4.12	0.0423
HDL-C, Median (IQR), mmol/L	1.24(0.32)	1.26(0.38)	0.13	0.7179
LDL-C, Median (IQR), mmol/L	3.18(1.37)	2.98(1.11)	5.11	0.0238
Degree of education, n (%)			7.23	0.0072
Primary school education	78(3.6)	1468(67.9)		
Junior middle school education	14(0.6)	520(24.0)		
High school and more than high school education	1	82(3.8)		
Frequency of exercise, n (%)			17.22	< 0.001
≥4 times/week	20(0.9)	1708(79)		
<4 times/week	73(3.4)	362(16.7)		

*IQR* interquartile range, *SD* standard deviation, *BMI* body mass index, *TC* total cholesterol, *TG* triglyceride, *HDL-C* high-density lipoprotein cholesterol, *LDL-C* low-density lipoprotein cholesterol, *SBP* systolic blood pressure, *DBP* diastolic blood pressure

### Gene-based model: SNPs associated with T2DM

Elastic net penalization allows for variable selection by shrinking the coefficients of the variables not related to the response to zero. Thus, variables with non-zero coefficients are considered as important predictors. Selection of the shrinkage parameter (lambda) for the elastic net model was performed by 20 repetitions of 10-fold cross-validation. The one-standard-error rule was used. Using this value as the minimum lambda value resulted in 5 variables being included in the prognostic model.

**Fig. 2:**
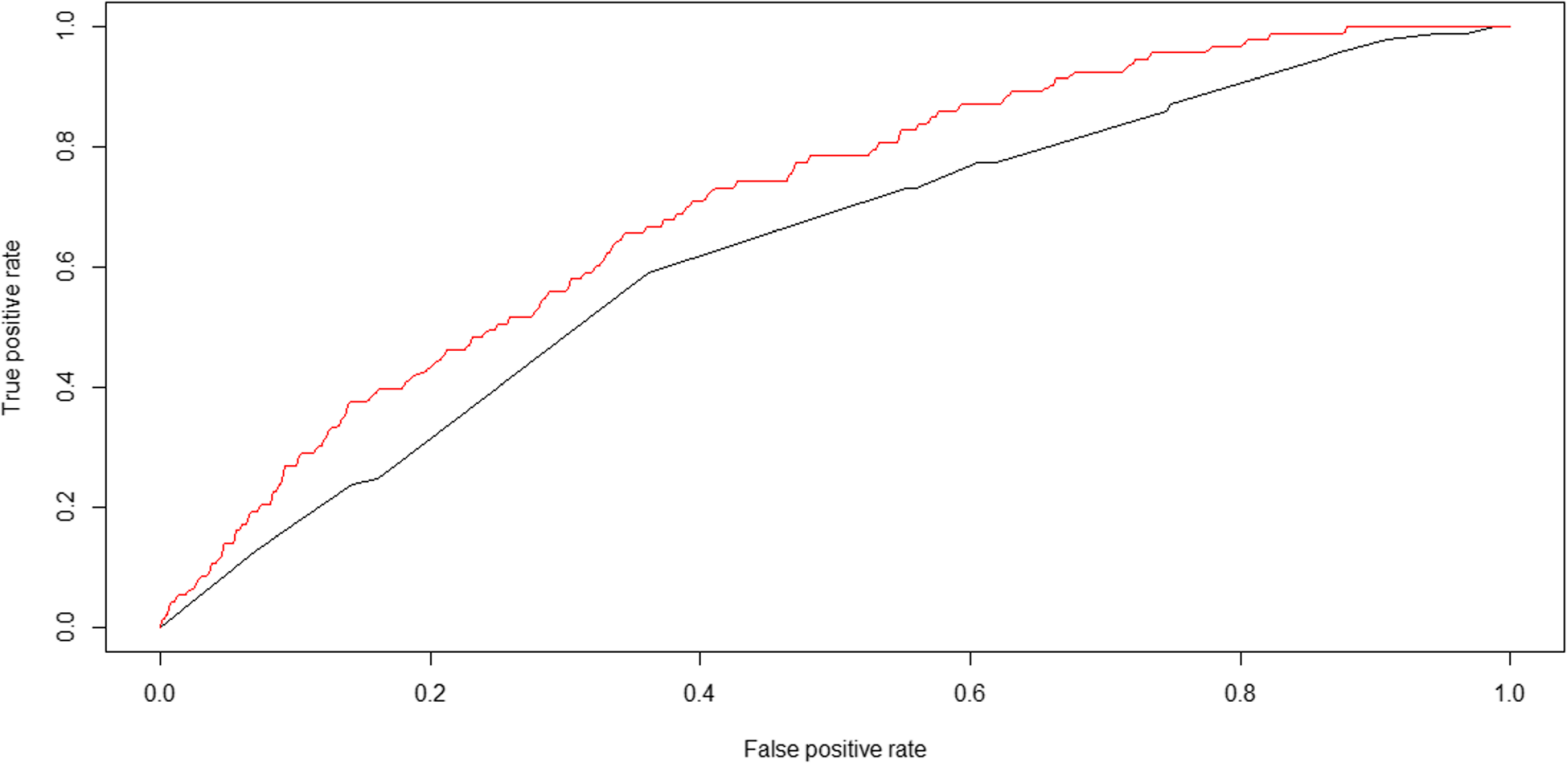
ROC curves of model A and model B: The black line represents model A; The red line represents model B

Initially, 102 SNPs were reduced to 5 potential predictors in 2163 people, and were features with nonzero coefficients in the elastic net model (Model A). The 5 potential SNPs were rs5805 in SLC12A3, rs12654264 in HMGCR, rs2065412 and rs414936 in ABCA1, and rs964184 in ZPR1. The area under the ROC curve for model A was 0.63 (Fig. 2). Figure 2 shows the ROC curves generated for each model. The black line represents model A, which was generated from SNP features using elastic net regression.

Table 2 is the association between the 5 SNPs and environmental factors with T2DM, which was examined under each gene model. Without adjustment, the recessive models of rs12654264 and dominant model of rs2065412 and rs4149336 were found to be significantly associated with T2DM (Table 2). In the additive models, the TT genotype of rs12654264 and CT genotype of rs4149336 were associated with a reduced risk of T2DM (unadjusted OR = 0.45, 95%CI = 0.24–0.84, *p* = 0.012; unadjusted OR = 0.59, 95%CI = 0.37–0.92, *p* = 0.019). Subjects carrying the TT genotype in the recessive model of rs12654264, CC + CT genotype in the dominant model of rs2065412, and TT + CT genotype in the dominant model of rs4149336 showed a lower risk of CAD than those with the AT+AA genotype, TT genotype, and CC genotype (unadjusted OR = 0.53, 95%CI = 0.32–0.90, *p* = 0.019; unadjusted OR = 0.30, 95%CI = 0.11–0.81, *p* = 0.018; unadjusted OR = 0.57, 95%CI = 0.38–0.87, *p* = 0.009).

**Table 2:** Associations of genetic models with risk of type 2 diabetes mellitus

SNP	Genotype	Unadjusted OR(95%CI)	Unadjusted P	Adjusted OR(95%CI)	Adjusted ***P***
Rs5805					
Additive	CT/TT	0.73(0.47 ~ 1.13)	0.155	0.71(0.46 ~ 1.10)	0.130
	CC/ TT	0.34(0.10 ~ 1.09)	0.069	0.34(0.11 ~ 1.12)	0.077
Dominant	CC + CT/TT	0.67(0.44 ~ 1.02)	0.062	0.66(0.43 ~ 1.01)	0.050
Recessive	CC/CT + TT	0.39(0.12 ~ 1.23)	0.107	0.40(0.12 ~ 1.28)	0.120
Rs12654264					
Additive	AT/AA	0.79(0.48 ~ 1.30)	0.347	0.81(0.49 ~ 1.35)	0.414
	TT/AA	0.45(0.24 ~ 0.84)	0.012	0.46(0.25 ~ 0.87)	0.017
Dominant	TT + AT/AA	0.66(0.41 ~ 1.06)	0.087	0.68(0.42 ~ 1.10)	0.512
Recessive	TT/AT+AA	0.53(0.32 ~ 0.90)	0.019	0.53(0.32 ~ 0.91)	0.021
Rs2065412					
Additive	CT/TT	0.31(0.11 ~ 0.84)	0.022	0.31(0.11 ~ 0.86)	0.299
	CC/TT	–	0.999	–	0.999
Dominant	CC + CT/TT	0.30(0.11 ~ 0.81)	0.018	0.73(0.48 ~ 1.10)	0.020
Recessive	CC/CT + TT	–	0.999	–	0.999
Rs4149336					
Additive	CT/CC	0.59(0.37 ~ 0.92)	0.019	0.59(0.37 ~ 0.92)	0.021
	TT/CC	0.53(0.25 ~ 1.14)	0.103	0.52(0.24 ~ 1.19)	0.094
Dominant	TT + CT/CC	0.57(0.38 ~ 0.87)	0.009	0.57(0.37 ~ 0.88)	0.010
Recessive	TT/CT + CC	0.68(0.32 ~ 1.41)	0.300	0.66(0.31 ~ 1.39)	0.271
Rs964184					
Additive	CG/CC	1.31(0.84 ~ 2.03)	0.232	1.40(0.90 ~ 2.18)	0.140
	GG/CC	2.24(0.99 ~ 5.09)	0.055	2.38(1.03 ~ 5.53)	0.043
Dominant	GG + CG/CC	1.41(0.93 ~ 2.13)	0.111	1.50(0.98 ~ 2.30)	0.060
Recessive	GG/CG + CC	2.21(0.91 ~ 4.52)	0.085	2.10(0.93 ~ 4.79)	0.076

Adjusted for waist circumference, the history of hypertension, the intake of meat, degree of education, exercise. The meaning of “/” is “VS”; the meaning of “+” is “and”

### All covariance-based model

Considering that model A only focused on the influence of genes on CAD, we recreated model B that included genetic characteristics and physiological, biochemical, and lifestyle indicators to identify factors related to CAD. When 102 SNPs were reduced to 5 potential predictors, the features of the 5 SNPs were presented in the gene-score calculation formula by elastic net. A gene-score was calculated for every person by linear combination of the selected features weighted by their respective coefficients. The gene-score was combined with 31 lifestyle variables, and 6 variables with gene-scores with nonzero coefficients were screened out by elastic net (Model B). The red line represents the model B generated from the gene-score and lifestyle features using the same technique. The area under the ROC for model B was 0.71 (Fig. 2). The 6 variables were gene-score, hypertension, meat intake, waistline, education degree, and exercise frequency (Table S2 and Table 3).

**Table 3:** Associations of gene-score and lifestyles with risk of type 2 diabetes mellitus

Characters	OR	95%CI	***P***
Gene-score	36.51	6.24–213.60	**< 0.001**
The history of hypertension	3.31	2.01–5.48	**< 0.001**
The intake of meat	1.50	0.98–2.31	0.06
Waist circumference	1.05	1.02–1.07	**< 0.001**
Degree of education	0.50	0.30–0.83	**0.007**
Exercise	0.80	0.66–0.98	**0.032**

The intake of meat, classified into never eat white meat,1–4 times every week, 5–7 times every week; > 7 times every week; *p* value < 0.05 was considered statistically significant

After adjusting for these 6 variables, the recessive models of rs12654264 and dominant models of rs2065412 and rs4149336 were still significantly associated with T2DM (adjusted OR = 0.53, 95%CI = 0.32–0.91, *p* = 0.02; adjusted OR = 0.73, 95%CI = 0.48–1.10, *p* = 0.02; adjusted OR = 0.54, 95%CI = 0.37–0.88, *p* = 0.01) (Table 2). In the additive models, the AA genotype of rs12654264, TT genotype of rs2065412, and CC genotype of rs4149336 still increased the risk of T2DM (Table 2). Table S2 is the Elastic net regularisation feature selection for gene-score and lifestyles.

### Interactions between gene polymorphism and other covariance estimators for the risk of T2DM

Considering that interactions may occur between variables in the model, we further explored these interactions through an extensive literature survey. At the same time, we had studied the correlation between the kinds of factors, for example, compared to individuals with lower genetic risk and healthy lifestyle, whether individuals with similar lifestyle but higher genetic risk have a higher starting risk of developing disease. Table 4 shows the effects of the interaction between 5 SNPs and hypertension on T2DM. In rs5805, rs12654264, rs4149336, and rs964184, compared to subjects without a history of hypertension carrying the non-risk genotype, those with a history of hypertension who carried the non-risk or risk allele were at a higher risk of T2DM (OR = 2.95, 95%CI = 1.38–6.30, *p* = 0.005; OR = 4.59, 95%CI = 2.22–9.49, *p* < 0.001; OR = 15.39, 95%CI = 2.04–116.30, *p* = 0.008; OR = 22.83, 95%CI = 3.15–165.69, *p* = 0.002). Although an interaction between the 5 SNPs and hypertension was not found (*p* values of RERI > 0.05), there was a cumulative effect in each model. For example, in rs5805, within the strata of TT, people with a history of hypertension had a higher risk of T2DM than those without a history of hypertension (OR = 3.60, 95%CI = 1.84–7.04, *p* < 0.001); in rs12654264, within the strata of AT+AA, compared to in people without a history of hypertension, those with a history of hypertension were at a higher risk of T2DM (OR = 2.68, 95%CI = 1.58–4.56, *p* < 0.001); in rs2065412, within the strata of hypertension, subjects carrying TT genotype had a higher risk of T2DM than those carrying the CC + CT genotype (OR = 1.94, 95%CI = 1.08–3.48, *p* = 0.026); in rs4149336, within the strata of hypertension, subjects carrying the CC genotype had a higher risk of T2DM than those carrying TT + CT; within the strata of the CC genotype, people with a history of hypertension were at a higher risk of T2DM (OR = 1.27, 95%CI = 1.00–1.61, *p* = 0.049; OR = 2.95, 95% CI = 1.53–5.68, *p* = 0.001) (Table 4).

**Table 4:** Interactions between Gene polymorphism and hypertension for the risk of type 2 diabetes mellitus

	Hypertension(−)		Hypertension(+)		OR(95%CI) for hypertension patients within strata of genotype	RERI (95%CI)	***p***
	case/control(n)	OR(95%CI)	case/control(n)	OR(95%CI)			
Rs5805							
Non-risk allele carriers (CC + CT)	9/503		29/549				
		1		2.95(1.38 ~ 6.30)	2.95(1.38 ~ 6.30)		
				*P* = 0.005	*P* = 0.005		
Risk allele carriers (TT)	11/482		44/536			1.35 (−0.60 ~ 3.32)	0.175
		1.28(0.52 ~ 3.11)		4.59 (2.22 ~ 9.49)	3.60 (1.84 ~ 7.04)		
		*P* = 0.592		*P* < 0.001	*P* < 0.001		
OR(95%CI) for risk allele carriers within strata of hypertension		1.28(0.52 ~ 3.11)		1.25 (0.98 ~ 1.59)			
		*P* = 0.592		*P* = 0.074			
Rs12654264							
Non-risk allele carriers (TT)	1/305		17/337				
		1		15.39 (2.04 ~ 116.30)	15.39 (2.04 ~ 116.30)		
				*P* = 0.008	*P* = 0.008		
Risk allele carriers (AT+AA)	19/680		56/748			−0.08 (−10.78 ~ 10.63)	0.989
		8.52 (1.14 ~ 63.95)		22.83 (3.15 ~ 165.69)	2.68 (1.58 ~ 4.56)		
		*P* = 0.037		*P* = 0.002	*P* < 0.001		
OR(95%CI) for risk allele carriers within strata of hypertension		8.52 (1.14 ~ 63.95)		1.48 (0.85 ~ 2.59)			
		*P* = 0.037		*P* = 0.165			
Rs2065412							
Non-risk allele carriers (CC + CT)	1/122		3/151				
		1		2.42 (0.25 ~ 23.60)	2.42 (0.25 ~ 23.60)		
				*P* = 0.446	*P* = 0.446		
Risk allele carriers (TT)	19/863		70/934			5.11 (−3.79 ~ 14.00)	0.260
		2.69 (0.36 ~ 20.24)		9.14 (1.26 ~ 66.42)	3.40 (2.03 ~ 5.70)		
		*P* = 0.338		*P* = 0.029	*P* < 0.001		
OR(95%CI) for risk allele carriers within strata of hypertension		2.69 (0.36 ~ 20.24) *P* = 0.338		1.94 (1.08 ~ 3.48) *P* = 0.026			
Rs4149336							
Non-risk allele carriers (TT + CT)	8/571		32/605				
		1		3.78 (1.73 ~ 8.26)	3.78 (1.73 ~ 8.26)		
				*P* = 0.001	*P* = 0.001		
Risk allele carriers (CC)	12/414		41/480			1.26 (−1.40 ~ 3.91)	0.353
		2.07 (0.84 ~ 5.11)		6.10 (2.83 ~ 13.13)	2.95 (1.53 ~ 5.68)		
		*P* = 0.115		*P* < 0.001	*P* = 0.001		
OR(95%CI) for risk allele carriers within strata of hypertension		2.07 (0.84 ~ 5.11) *P* = 0.115		1.27 (1.00 ~ 1.61) *P* = 0.049			
Rs964184							
Non-risk allele carriers (CC)	10/615		41/690				
		1		3.65 (1.82 ~ 7.36)	3.65 (1.82 ~ 7.36)		
				*P* < 0.001	*P* < 0.001		
Risk allele carriers (GG + CG)	10/370		32/395			0.67 (−1.72 ~ 3.05)	0.584
		1.66 (0.69 ~ 4.03)		4.98 (2.42 ~ 10.25)	3.00 (1.45 ~ 6.18)		
		*P* = 0.261		*P* < 0.001	*P* = 0.003		
OR(95%CI) for risk allele carriers within strata of hypertension		1.66 (0.69 ~ 4.03) *P* = 0.261		1.17 (0.92 ~ 1.48) *P* = 0.204			

*p*-value < 0.05 is considered statistically significant

Table 5 shows the effect of the interaction between meat intake, exercise frequency, dyslipidemia, and hypertension on T2DM. For meat intake, compared to in people without hypertension who eat white meat less than three times per week, those with hypertension who eat meat were at a higher risk of T2DM regardless of the number of times per week (OR = 4.15, 95%CI = 2.08–8.29, *p* < 0.001; OR = 5.60, 95%CI =2.68–11.7, *p* < 0.001). Within the strata of hypertension, people who eat white meat more than three times per week had a higher risk of T2DM than people who eat white meat less than three times per week (OR = 2.49, 95%CI = 1.18–5.22, *p* = 0.016). For the frequency of exercise, compared to in those without hypertension who had a good exercise habit (≥4 times/week), those with hypertension who did more or less exercises were at a higher risk of T2DM (OR = 5.16, 95%CI = 1.51–17.67, *p* = 0.009; OR = 79.55, 95%CI = 24.64–256.97, *p* < 0.001). Within the strata of hypertension, people who exercised less than 3 times per week had a higher risk of T2DM than those who exercised less than 4 times per week; additionally, within the strata of those who exercised less than 4 times per week, people with hypertension had a higher risk of T2DM than people without hypertension (OR = 15.42, 95%CI = 8.77–27.12, *p* < 0.001; OR = 2.95, 95%CI = 1.65–5.27, *p* < 0.001). Compared to subjects without dyslipidemia or hypertension, those who had dyslipidemia only, hypertension only, or two diseases at the same time were at a higher risk of T2DM (OR = 12.26, 95%CI = 3.66–41.06, *p* < 0.001; OR = 5.59, 95%CI = 1.63–19.19, *p* = 0.006; OR = 10.43, 95%CI = 3.23–33.64, *p* < 0.001). Interactions between the 3 models were not detected (*p* values of RERI > 0.05) (Table 5).

**Table 5:** Interactions between other lifestyles and hypertension for the risk of type 2 diabetes mellitus

	Hypertension(−)		Hypertension(+)		OR(95%CI) for hypertension patients within strata of other lifestyles	RERI (95%CI)	***p***
	case/control(n)	OR(95%CI)	case/control(n)	OR(95%CI)			
The intake of meat							
<3times/week	10/682		46/756				
		1		4.15 (2.08 ~ 8.29)	4.15 (2.08 ~ 8.29)		
				*P* < 0.001	*P* < 0.001		
≥3times/week	10/303		27/329			0.20 (−2.74 ~ 3.13)	0.896
		2.25 (0.93 ~ 5.46)		5.60 (2.68 ~ 11.7)	1.35 (0.82 ~ 2.21)		
		*P* = 0.073		*P* < 0.001	*P* = 0.234		
OR(95%CI) for people who eat much more meat within strata of hypertension		2.25 (0.93 ~ 5.46)		2.49 (1.18 ~ 5.22)			
		*P* = 0.073		*P* = 0.016			
The frequency of exercise							
≥4times/week	3/489		24/319				
		1		5.16 (1.51 ~ 17.67)	5.16 (1.51 ~ 17.67)		
				*P* = 0.009	*P* = 0.009		
<4times/week	17/496		49/766			48.38 (−11.90 ~ 108.66)	0.116
		26.98 (7.82 ~ 93.07)		79.55 (24.64 ~ 256.97)	2.95 (1.65 ~ 5.27)		
		*P* < 0.001		*P* < 0.001	*P* < 0.001		
OR(95%CI) for people who eat much more meat within strata of hypertension		26.98 (7.82 ~ 93.07)		15.42 (8.77 ~ 27.12)			
		*P* < 0.001		*P* < 0.001			
Dyslipidemia							
Dyslipidemia (−)	3/489		24/319				
		1		12.26 (3.66 ~ 41.06)	12.26 (3.66 ~ 41.06)		
				*P* < 0.001	*P* < 0.001		
Dyslipidemia (+)	17/496		49/766			−6.43 (−17.08 ~ 4.22)	0.237
		5.59 (1.63 ~ 19.19)		10.43 (3.23 ~ 33.64)	1.87 (1.06 ~ 3.28)		
		*P* = 0.006		*P* < 0.001	*P* = 0.03		
OR(95%CI) for dyslipidemia patients within strata of hypertension		5.59 (1.63 ~ 19.19)		0.85 (0.51 ~ 1.41)			
		*P* = 0.006		*P* = 0.529			

*p*-value < 0.05 is considered statistically significant

## Discussion

To construct the model, 133 candidate features were reduced to 7 potential predictors by examining the predictor-outcome association by shrinking the regression coefficients using the elastic net method. This method not only is superior to the method of choosing predictors based on the strength of their univariable association with outcome [27–29], but also enables the panel of selected features to be combined into a model. Thus, the model, which makes use of easily accessible metrics, can serve as a more convenient biomarker for explaining T2DM.

As T2DM is a complex disorder, and several genes have been implicated in its etiology and evolution. The identification of risk alleles is useful because if the involved genes and their functions are known, this information can be used to develop prevention, treatment, prognosis prediction, and/or curative methods for the disease. In the gene-based model, we examined the influence of genetic polymorphisms in four genes (SLC12A3, HMGCR, ABCA1, ZPR1) on T2DM through elastic net screening. Our data demonstrated that rs5805 in SLC12A3, rs12654264 in HMGCR, rs2065412 and rs414936 in ABCA1, and rs96418 in ZPR1 were significantly associated with T2DM.

We found that the minor allele (“C”) of rs5805 in SLC12A3 was associated with a reduced risk of T2DM in the Chinese population. SLC12A3, located on 16q13, encodes a thiazide-sensitive Na + Cl– cotransporter that mediates reabsorption of Na + and Cl– in the renal distal convoluted tubule and is expressed specifically in the kidneys [30]. Studies of SLC12A3 suggested that its genetic variants and rare mutations impact the development of hypertension and T2DM and/or nephropathy in Asian populations [31–33], which is consistent with the results of our study.

Our finding that variants in HMGCR were associated with the risk of diabetes. People carrying the TT genotype of rs12654264 are at a reduced risk of T2DM. Past studies have shown that, HMGCR variants are associated with obesity or its subphenotypes, such as weight, BMI, or waist circumference [34–36]. Thus, the mechanism by which HMGCR variants increase the risk of diabetes is likely mediated by weight gain.

ABCA1 plays an important role in cholesterol metabolism, particularly for HDL-C [37]. Previous investigations have showed that the ABCA1 gene may influence cardiovascular risk in the general population [38]. In addition, the ABCA1 R230C polymorphism may play an important role in maintaining glucose-mediated insulin secretion, in turn, leads to a 4-fold increase occurrence of diabetes [39]. Few studies have examined the role of ABCA1 polymorphism (rs2065412 and rs414936) in diabetes. We found a significantly higher frequency of both the T allele and genotype in the control group compared to in patients, indicating that the T allele is a protective factor against diabetes mellitus.

ZPR1 is located ~ 1.6 kb upstream of the APOA5-A4-C3-A1 gene complex. We found that rs964184 of ZPR1 was significantly associated with T2DM in Chinese individuals. This is consistent with the results of a previous study [40, 41]. rs964184 is in the intron region of ZPR1 at chromosome 11q23.3. ZPR1 is an essential regulatory protein for cell proliferation and signal transduction and may have multiple physiological functions [41, 42].

Multiple environmental risk factors, including gender, personal fitness status, weight, other physical conditions, and their interactions, can modulate serum lipid profiles, in addition to the effects of genetic background [13, 43, 44]. In the present study, demographic characteristics and lifestyle factors of the participants, including waistline, education degree, exercise frequency, hypertension, and meat intake, influenced T2DM. This has been confirmed in previous studies [11–13, 43, 44].

Epidemiological experts have suggested that quantitative interactions in the additive model are best suited for assessing the importance of interactions [26]. RERI, as well as the *p* values and 95%CI of RERI, were determined in this study. The RERI caused by an interaction is generally considered as the standard measure of an additive model interaction in case-control studies. We explored the interactions of gene-lifestyle factors, gene- biochemical indicators, and certain lifestyle factors with the risk of T2DM. Although the interactions between these indices were not statistically significant, those carrying risk alleles of these SNPs who also had a history of hypertension or dyslipidemia were also at a high risk of disease.

This study had some limitations. First, our model was designed to explain the relationship between variables and disease and not to predict the risk of T2DM, and thus the model was not tested in new populations. Second, most responses related to lifestyles were obtained through questioning of the patients, and thus, there may have been recall bias. Finally, the conclusions may only be applicable to people in southern China. Studies in multiple regions and different populations using a randomized, large-scale, long-term design are needed.

## Conclusions

In conclusion, the model which we built showed that four SNPs and 5 variance-covariance estimators were associated with T2DM in people in southern China. These results will provide a theoretical basis for gene and risk factor screening to prevent T2DM.

## Supplementary information

**Additional file 1. **The following are available online at www.mdpi.com/xxx/s1, **Table S1.** The information of 107 SNPs. **Table S2.** Elastic net regularisation feature selection for gene-score and lifestyles.

## Data Availability

The datasets analysed during the current study are not publicly available due [the data is being further analyzed] but are available from the corresponding author L.Y. on reasonable request.

## Abbreviations

BCMO1: Beta-carotene monooxygenase 1
LDLR: Low-density lipoproteins receptor
PCSK9: Proprotein convertase subtilisin kexin type 9
SLC12A3: Solute carrier family 12 member 3
KCNJ1: Potassium voltage-gated channel subfamily J member 1
CAD: Coronary artery disease
GWAS: Genome-wide association studies
SNPs: Single-nucleotide polymorphisms
AIDS: Acquired immune deficiency syndrome
TC: Total cholesterol
BMI: Body mass index
HDL-C: High-density lipoprotein cholesterol
LDL-C: Low-density lipoprotein cholesterol
MAF: Minor allele frequency
