# Supplementary material for: Model of genetic and environmental factors associated with type 2 diabetes mellitus in a Chinese Han population
Source: BMC Public Health. 2020 Jun 29;20:1024. doi: 10.1186/s12889-020-09130-5 (PMC7325035; doi:10.1186/s12889-020-09130-5)
Supplement: Supplementary file 1 — Additional file 1. The following are available online at www.mdpi.com/xxx/s1, Table S1. The information of 107 SNPs. Table S2. Elastic net regularisation feature selection for gene-score and lifestyles. [file 12889_2020_9130_MOESM1_ESM.zip › TableS2R3.doc]

Table S2 Elastic net regularisation feature selection for gene-score and lifestyles

| Elastic net regularisation feature selection/model | Variable weighting/cofe |
| --- | --- |
| Gene-score | 0.5066 |
| The history of hypertension | 0.2693 |
| The intake of meat | 0.0181 |
| Waist circumference | 0.0130 |
| Degree of education | -0.0507 |
| Exercise | -0.0148 |
